# The ‘Real Without Law’ in Psychoanalysis and Neurosciences

**DOI:** 10.3389/fpsyg.2018.00851

**Published:** 2018-06-26

**Authors:** Adriano Aguiar

**Affiliations:** Mental Health and Family Medicine, Institute of Psychiatry, Psychiatric Center of Rio de Janeiro, Federal University of Rio de Janeiro, Rio de Janeiro, Brazil

**Keywords:** psychoanalysis, neurosciences, Lacan, real, second nature, cognitive science

## Abstract

In this article, we will examine some of Lacan’s concepts on the relation between psychoanalysis and science. The difference that Lacan states between the real for science, which would be entirely governed by laws, and the real for psychoanalysis – a ‘real without law’ – risk to lead to an irreducible separation between the two fields. However, as the article shows, that separation between psychoanalysis and science is not the position defended by Freud and Lacan. Indeed the latest discoveries in the field of neurosciences challenge the traditional conception of the real for science, bringing it closer to the real ‘without law’ that characterizes psychoanalysis. Conceiving the real for science as a real that is opened to contingencies and not entirely governed by laws, is the first necessary step for a new alliance between psychoanalysis and science.

## Introduction

Since the 1990s some important neuroscientists, such as Gerald Edelman, V. S. Ramachandran, Antonio Damasio and others, began to express their interest in approaching psychoanalysis and neurosciences. The studies conducted by these scientists led to the creation of the Neuropsychoanalysis Association, an international network of non-profit organizations that support the dialog between neurosciences and psychoanalysis.

Even if those more involved with Lacanian psychoanalysis tended to be more suspicious about an approximation between psychoanalysis and neuroscience, this scenario has been changing in the last decade since some Lacanian psychoanalysts and philosophers have done quite fruitful theoretical dialogs between some experimental findings in the fields of genetics and neurosciences and some theoretical concepts from Lacanian psychoanalysis (Ansermet and Magistretti, 2004; Zizek, 2008; Johnston, 2013).

This article seeks to contribute to the debate between Lacanian psychoanalysis and neurosciences, discussing a specific point of the conceptual underpinnings of Lacanian theorizations about science. If the difference that Lacan states between the real for science, which would be entirely governed by laws, and the real for psychoanalysis – a ‘real without law’ – risk to be seen as the basis for an irreducible separation between the two fields, we tried to show that the complete separation between psychoanalysis and science was never the position defended by Freud and Lacan. We argue that the latest discoveries in the field of neurosciences challenge the traditional conception of the real for science, bringing it closer to the real ‘without law’ that characterizes psychoanalysis, what makes possible a entirely new kind of dialog between the two fields, since the idea of the real that arises from contemporary neurosciences is a real that is opened to contingencies and not entirely governed by laws.

## Psychoanalysis and Natural Sciences

The development of psychoanalytical theory by Freud was often associated with some concepts or metaphors that he took from the natural sciences, which exerted a strong influence in his thinking. Otherwise, Lacan’s inspiration in Levi-Strauss and Saussure’s structuralism, contributed decisively to give Lacanian psychoanalysis its own epistemological vocabulary that moved away from naturalistic influences (Bezerra, 2013). Although Lacan had been interested in animal behavior and biology in the early days of his teaching, this interest was in the opposite direction of the naturalism present in some post-Freudian psychoanalysts thinking. Differently from the adaptationist perspective present in the naturalistic inspiration of most post-Freudian authors, for Lacan human subjectivity is something that emerges as the effect of the symbolic order, constituting a break from the immediate plan of nature.

However, if Lacan’s teaching was clearly marked by an anti-naturalistic perspective, we could not say that he sustains that psychoanalysis should have an anti-scientific stance. We know that Freud thought that the worldview (Weltanschauung) of psychoanalysis should be no other than science’s one. At the end of the conference The question of a Weltanschauung Freud states that: “In my opinion, psychoanalysis is incapable of creating a Weltanschauung of its own. Psychoanalysis does not need one; it is part of science and can adhere to the scientific Weltanschauung” (Freud, 1933 p. 89).

In the same direction, Lacan is emphatic in reproving those who claim that psychoanalysis should stand in a position of exteriority regarding the scientific field: “We say, contrary to what was invented on an alleged breaking of Freud with the scientism of his time, that it was this same scientism (...) which led Freud, as is shown in his writings, to open the way that will forever have his name. We say that this way has never left the ideals of this scientism since it is called like that and that the mark it brings form it is not contingent, but essential” (Lacan, 1966/1998, p. 871).

For Lacan, the relationship between psychoanalysis and science rests on the epistemological discontinuity produced by the emergence of modern science which promoted a radical transformation in the modern conception of the subject that was essential for the emergence of psychoanalysis. The Cartesian cogito is considered by Lacan a key correlate to the emergence of modern science, which he characterizes as a moment of rejection of all traditional knowledge in order to establish a “grounding in being” for the subject. A subject, emptied of knowledge, devoid of content and representation, without substantial density, which only exists in the act: “I think, therefore I am.” This is the subject of science. And for Lacan this subject is none other than the subject upon which psychoanalysis itself operates: “That is why it was important to promote, first and foremost, and as a fact to be distinguished from the question of whether psychoanalysis is a science (if your field is scientific), just the fact that its practice does not imply another subject than the subject of science” (Lacan, 1966/1998, p. 878).

## The Real for Science and the Real for Psychoanalysis

For Lacan, psychoanalysis would not be possible without the advent of modern science. Following Koyré (1957/2010), Lacan thinks that science is always based on the assumption that there is “knowledge in the Real”. The “laws of nature” implies that there is something like an articulated network of “signifiers” that are present in the Real, and, at least in the early part of his teaching, Lacan searched for a rapprochement between psychoanalysis and the scientific field based on this assumption that characterizes the foundation of science: that there is knowledge in the Real. Indeed, at the beginning of his teaching, Lacan seeks an alignment between psychoanalysis and science via structuralism. According to Jacques-Alain Miller (Miller, 2002a), Lacan wandered of being able to suspend the segregation of psychoanalysis by science trying to translate the tragic aspect of human experience into the mathematics. That’s why Lacan was so attracted to some kind of mathematization of psychoanalytic experience. In this perspective, the unconscious is constituted by signifiers organized according to the laws of language, which operate independently of the subject’s consciousness. The subject itself is an effect of the functioning of these laws. That is why the analyst can rely on the Freudian free association. It certifies that for psychoanalysis there is the assumption that there is a certain “knowledge in the Real”. This is a deterministic assumption of psychoanalysis: speak the patient whatever she says, the unconscious signifiers will emerge in her speech, for there are laws of language that determine the unconscious functioning. In this sense, we could say that there is a kind of “knowledge in the Real” which is present in the analytical experience, it’s not just about hermeneutics or narratives (Miller, 1989).

Towards the end of his teaching, Lacan will gradually replace the causal language that marked his beginnings to isolate a break in the chain of determination, stating that the real with which we deal in the analytic experience is a real that conveys the absence of law – a “real without law”. It is a real that is not based on the positive axiom that for Lacan characterizes science – “there is knowledge in the real” – but a real that rests in contingency and in the absence of sexual relation (“il n’y a pas de rapport sexuel”): “Throughout his teaching, ready to confront the discourse of science on their own ground, he [Lacan] adopted a causal language. Until he found a break in the causality, a break in determination, finding, synthesizing – why not say that? – certain results under the species of: “there is no sexual causality”. He said “relation”. But he said relation meaning that there is no causality. There is no law of the relation between the sexes. He thought that by this way he could oppose to the real for science – which is a real containing knowledge – to the real of psychoanalysis - under the species of a real that does not contain a knowledge and that would convey the knowledge of the unconscious. It would convey, first of all, especially the absence of law, precisely the hole in this knowledge. “There is no sexual relation” is the notion of the absence of law. The sexual law cannot be written. It is then that the term contingency becomes the master word, rather than cause” (Miller, 2012).

## Psychoanalysis and Science: Three Logical Moments

In separating these two conceptions of the real, the real for science and the real for psychoanalysis, Lacan highlights the specificity of psychoanalysis, which can then definitely give up the aim of being recognized as part of the scientific field. On the other hand, this doesn’t imply that we should believe that psychoanalysis and science are two completely separated fields, with nothing to say to each other. According to Miller (2011), we can divide into three logical moments the relationship between psychoanalysis and science in Lacan’s teaching. In the beginning, Lacan states that psychoanalysis depends on science, putting scientific knowledge as a condition of possibility for the emergence of psychoanalysis. In a second stage, Lacan moves a little from this perspective, understanding that psychoanalysis finds its place out of a failure or incompleteness in the discourse of science. Miller (ibid) notes that in the Italian Note (Lacan, 1973/2003), Lacan says that the very functioning of the discourse of science tends to produce an effect of reaction or protest that Lacan calls “humanist.” This reaction aims to highlight that the knowledge in the real does not account for everything and that what is essential in the humankind is not the scientific objectivity, but rather something that always escapes from the scientific knowledge. The “humanist protest” claims for what Lacan qualifies as “docta ignorantia” (learned ignorance) against the scientific knowledge and, in a certain way, psychoanalysis finds its place in this humanism protest since she seeks to reintroduce the dimension of the subject that the discourse of science tends to suture. Psychoanalysis then appears as a “waste product” of the discourse of science and the psychoanalyst is called to challenge the discourse of science reintroducing the dimension of the subject.

Miller (2011) retakes this proposition of Lacan to show that he didn’t stop there and to state that the position of psychoanalysis in relation to science should not stay in this humanist protest neither refuse the scientific knowledge. Miller points out that, still in the Italian Note, by introducing the desire to know that characterizes psychoanalysis, Lacan would point to a third logical moment in this circuit, in which psychoanalysis is not situated in the field of humanistic protest against science, but embodies the return of the science in the field of the learned ignorance. The desire to know that characterizes the position of the psychoanalyst, should not be confused with the learned ignorance, neither with the scientific knowledge. Psychoanalysis deals, otherwise, with something that is transmitted from science when the scientific desire enters the humanistic field of the learned ignorance. The desire to know that characterizes psychoanalysis thus entails a paradox: it is an effect of science without being, however, according to the scientific desire. It is an unprecedented desire to know that has its roots in science, but should not be confused with the scientific desire either with the denial of the scientific knowledge. The psychoanalytical stance is not a scientific one, but this doesn’t imply that it refuses the knowledge in the real. It’s a position that takes in charge the issues of “truth” with the means of science.

For Miller, the core of Freud’s ambition is the return of the question about the truth within the scientific field: “We note that for the defenders of the scientific discourse, far from being distinguished from the learned ignorance, [psychoanalysis] is mistaken for it. Psychoanalysis may seem a reprint of the learned ignorance, which was as Lacan showed her for a while. On the other hand, for the learned ignorance partisans, the humanists, psychoanalysis seems to be attached to the values of science. This dual position led Lacan to assert that science (he said this with the best reason in the world, that is, with the example of Freud) inspired in some dissatisfied with the learned ignorance, with the humanistic knowledge, the desire to treat the truth in an unprecedented way. Lacan then speaks of a desire to know, which is the transformation of the desire of science when it finds what was excluded and foreclosure: the problem of truth” (Miller, 2011, p. 414).

## From the Psychoanalytic Protest to a New Alliance With Science

The discovery of the hysterical symptom by Freud was made in the context of the scientific discourse, it had incidence over a real in the scientific sense, a Galilean real, a real that accommodates knowledge. But Freud introduces something new there, postulating that there is a sense in the real, that the symptom has a meaning. For this, Miller (2005) states that psychoanalysis has emerged as a “corruption of scientific knowledge,” as for science there is knowledge in the real, but it doesn’t mean to “say” anything. Postulate that there is sense in the real implies that it means something, that there is something like a certain “intentionality” of the symptom which is the condition of possibility for analytic interpretation: “the meaning in the real is the support of the symptom in the analytical perspective” (Miller, 2005, p. 15).

Throughout the twentieth century, it was widely accepted this conception of the symptom – the analytic symptom. The symptom was seen as a mental symptom, as an unconscious symptom, a symptom that has a meaning to be interpreted. Nowadays, however, the situation has changed. After the DSM-III and the strong influence of biology in the mainstream psychiatric discourse (Aguiar, 2004), the symptom has been seen, in this perspective, as a sign of some disorder of the brain, as something absolutely meaningless, whose physiological causes neurosciences would explain.

How to answer to this from a psychoanalytic point of view? Miller (2005) states that there are three answers today: first, there are those who wish to adhere to the knowledge in the real, that is, those who believe that psychoanalytic concepts could be translated into the language of the neurosciences. Then there is the opposite position, the psychoanalytic protest which, according to Miller, is appealing, but in vain. This protest consists in refusing the knowledge in the real. For Miller, however, Lacanian’s position can’t be the refusal of the scientific knowledge. Lacanian psychoanalysts must admit that there is knowledge in the real, but at the same time, formulate that there is a hole in this knowledge, that sexuality makes a hole in the knowledge in the real. In this sense, Miller proposes a position that is more compatible with a certain alliance with science, a “new alliance” with science that takes into account the real at stake in psychoanalysis: “We can say that here we find the index that points to what Lacan brings and that doesn’t consist absolutely in a refusal of the scientific real and of the knowledge in the real. Because deny the scientific real and refuse the discourse of science is a road to perdition that opens the way to all intrigues in the ‘psi’ field. Intrigue is not an injurious term. Do not refuse that knowledge, admit that there is knowledge in the real, but at the same time formulate that in this knowledge there is a hole, that sexuality makes a hole in this knowledge. It is a transformation of Freud, no doubt. It’s a new alliance between psychoanalysis and science, if I dare to say, that rests on the non-relation. The non-relation gives the site of Lacanian practice. This should be understood as follows: the “sexual non-relation” is what makes a balance with the statement that says that “there is knowledge in the real”. It is sexuality that makes an objection to the omnipotence of the discourse of science” (Miller, 2005, p. 16).

Miller doesn’t say much more about how could be such new alliance between science and psychoanalysis. This seems to be a task to be done, with which this article aims to contribute. But it is important to notice that he writes these lines after pointing out the shift in the prevailing conception of the symptom in the contemporary era since the neuroscientific discourse became hegemonic. So when he speaks about science here, he doesn’t seem to be talking about physics or mathematics as is more usual within the Lacanian field, but about the natural sciences and more specifically the neurosciences.

In another passage of the same text – which is the transcript of one of Miller’s conferences at the Congress of the World Association of Psychoanalysis in 2004 – Miller draws the attention of the psychoanalysts, saying that one should not “insult the future” and proposes a certain openness to what happens in the field of neuroscience and cognitive sciences: “Freud’s metapsychology showed signs of weakness, in the second half of the twentieth century. And we could say that Lacan proceeded a logical-linguistic translation of that metapsychology. He himself acknowledged having to go through this in order to give a breath of life to psychoanalysis. So it is not absurd, *a priori*, to try to give a neurocognitive translation of the metapsychology. We can say that it will be judged by its results” (Miller, 2005, p. 11).

We propose to take seriously these indications from Miller: how to think about a new relationship between psychoanalysis and neuroscience that rest on the non-relation? How to conceive the relation between psychoanalysis and neuroscience in a way that doesn’t deny the scientific knowledge, without ever losing sight of what makes a hole in this knowledge?

Jean-Claude Milner, which is another important reference in the field of Lacanian psychoanalysis, especially regarding the relationship between psychoanalysis and science, proposes in one of his recent books (Milner, 2011), that to think about the relation of Lacanian psychoanalysis with the science of today, you may need to move away from Koyré’s classical perspective that privileges the mathematization of the real. According to Milner, when Galileo wrote that nature was written in mathematical letters, the strong word should be “letter” and not mathematics. Because the essence of the Galilean turn would be, in Milner’s opinion, the literalization, of which mathematics is just one way among others.

According to Milner, the position of Lacan with respect to science was deeply marked by the science of his time, which was a science dominated by the physics of Newton and Einstein. Today, however, for Milner that physics is dead, and to approach the new paradigms of science one would have to consider genetics and the biological sciences instead. And for Milner, the current biological science is not mathematized but literalized. The type of literalization scientists dedicated to the transcription of the genetic code held, for example, may even involve certain calculations, but do not constitute a mathematization. According to Milner, a kind of literalization of the living is emerging in biological sciences, and take it seriously would compel the Lacanian position in relation to science to change, especially as regards the reference to Koyré: “On the back cover that he wrote to the book Autres Écrits, Jacques-Alain Miller acknowledged in the decoding of the genome a ‘promise of new marriage of the signifier with the living’. But overall, I have sometimes the impression that we don’t take into account the passage of the years. In 1965, in The science and the truth, Lacan could write that ‘Koyré is our guide’. Almost half a century has passed, the epistemology and the history of science have changed, and repeated offensives against Koyré’s model were done, which the most decisive ones were not always recognized; as Foucault’s in The Order of Things. Determinate what remains of Koyré should be a matter to consider. I didn’t judge opportune to express myself in detail – neither on what remains or not from Koyré in general, nor on what in Lacan could be affected by an eventual obsolescence of Koyré. In L’oeuvre Claire I had put myself where it was placed by Lacan himself, namely, in a space where the supreme science is the mathematical physics. I haven’t addressed the question of biology” (Milner, 2011, p. 19).

In 1984, Prigogine and Stengers (1997) had already pointed out that the classical model of science, as Koyré interpreted it, implied a conception of nature as an “automaton nature”, entirely governed by mechanical laws accessible to human’s rationality through the laws of mathematics. In fact, much of the natural phenomena respond to this model, as the dynamics of celestial bodies and the law of gravity, which obey the laws that mathematics succeed to identify. But the scientific discoveries of the twentieth century require a transformation in our conception of nature that has not been fully assimilated into the culture, which implies a perception of nature not as something stable, fully governed by predictable and immutable laws, but as a nature that is open for transformation: “Modern science was established as a product of a culture, against certain dominant conceptions of this culture (Aristotelianism in particular, but also the magic and alchemy). One could even say that it was constituted against nature itself because it denied her complexity and becoming on behalf of an eternal and knowable world ruled by a small number of simple and immutable laws” (Prigogine and Stengers, 1997, p. 4).

As stated by these authors for over 30 years, today’s science is no longer the classical science, and those who continue to conceive nature as an automaton governed by mechanical and predictable laws are losing sight of what matters most to the science of our time, i.e., not the continuities and stable situations, but developments, crises and instabilities, which require another conception of naturalism.

We understand that to conceive of a “new alliance” between psychoanalysis and science, which rests on the “non-relation,” i.e., to conceive a relation between psychoanalysis and science that take into account the “real without law” (Miller, 2002b) that guides the practice of Lacanian psychoanalysis, one must examine what is happening in the field of contemporary natural sciences, particularly in the field neurosciences, so that you can seek in this field if it’s possible to find some openings for the contingent real (Miller, 2008) which is proper of psychoanalysis.

## Neurosciences and the Real Without Law

In the book The Dappled World: A Study in the Boundaries of Science (Cartwright, 1999), the philosopher of science Nancy Cartwright shows that anyone who looks clearly and honestly at the current state of science will come across with the fact that we can no longer accept the often assumed equivalence between scientific realism and universality of the laws. The author notes that in the various domains covered by science we are increasingly faced with the fact that we can’t establish universal laws: “The kind of knowledge we can defend from our impressive scientific successes does not point to a unified world from of a universal order, but to a dappled world of stained objects” (Cartwright, 1999, p.10).

As Adrian Johnston (Johnston, 2011) argues, it is not necessary to go down into the world of quantum physics to give scientific status to the indeterminacy that is characteristic of human subjectivity. According to Johnston, a veritable avalanche of current research in genetics and neuroscience reveals that brains and human bodies are much less determined by pre-established rules than we previously thought. The biomateriality of nature establishes a relatively small number of limiting parameters for the living being. It hardly functions as something that determines all the details of life. The biologist Francisco Varela and his collaborators (Varela et al., 1991), for example, describe the ontogenetic and phylogenetic unfolding of living beings as satisfactory/sufficient processes that work only to achieve what is good enough to survive, for long enough to reproduce. Evolution doesn’t compel the production of what would be Ideal, allowing the arising of a great diversity at all levels of interaction with the environment, and may even permit the persistence of highly dysfunctional gaps in life: “The second step, then, is to analyze the evolutionary process as having a solution that is satisfactory rather than optimal: in this perspective [natural] selection functions as a broad survival filter that admits any structure that has enough integrity to persist. In this point of view, the focus of the analysis is no longer directed at the characteristic features, but rather at the patterns of organisms, via their life history. Another metaphor recently suggested for this post-Darwinian conception of the evolutionary process is evolution as bricolage, the joining of parts and elements into complex matrices, not because they fulfill some ideal project, but simply because they are possible. Here the evolutionary problem is no more like forcing a precise trajectory of the requirements of ideal aptitude; it is rather how to prune the multiplicity of viable paths that exist at any point” (Varela et al., 1991, p. 196).

The French philosopher Catherine Malabou (2008) develop the philosophical issues that arise with the discovery of neuroplasticity. For her, this discovery entails that we have to acknowledge that human brain is organized and reorganized “dialectically,” continually suffering multiple oscillations between its “malleable flexibility” and its “resistant fixity.” Catherine Malabou states that we have not yet fully assimilated the results of the revolutionary discoveries made by neurosciences in the last 50 years. In particular, we have not yet assimilated the importance of the constitutive historicity of the brain, which is implicated in the discovery of brain plasticity: “Our brain is plastic, and we do not know it. We are completely ignorant of this dynamic, this organization, and this structure. We continue to believe in the “stiffness” of a fully genetic brain” (Malabou, 2008. p. 4). For Malabou, the relatively recent discovery of brain neuroplasticity should change our conception of the brain as entirely predetermined by the laws of nature, allowing us to see the brain as something modifiable, “formable” and formative at the same time. Malabou identifies three levels in which cerebral neuroplasticity operates: (1) in the modeling of neural connections during embryonic and childhood development; (2) in the modification of the neuronal connections that occur through the plasticity of the synaptic modulation to lifelong learning; and (3) the ability to repair after some kind of injury.

In the book Le cerveau intime (Jeannerod, 2002), Marc Jeannerod argues that if a synapse belongs to a circuit often used, it tends to increase in volume, permeability, and effectiveness. On the contrary, if a synapse is poorly used it tends to become less effective. According to Jeannerod, it is a biological mechanism of individuation that makes each brain unique: “The theory of synaptic efficacy allows us to explain the gradual molding of a brain under the influence of individual experience, to the point of it is possible for us in principle to account for the individual characteristics and particularities of each brain. We are dealing here with a mechanism of individuation that makes each brain a single object, despite its adherence to a common model” (Jeannerod, p.63).

The psychoanalyst François Ansermet and the neuroscientist Pierre Magistretti also emphasize this aspect of brain plasticity as a biological indicator of the uniqueness of the brain, which is shaped by experience. In the book, A chacun son cerveau (Ansermet and Magistretti, 2004) these authors argue that the recent discoveries of neurobiology demonstrate that the plasticity of the neural network allows the inscription of the lived experience in the brain. The traces are inscribed, associated and modified throughout life through the mechanisms of brain plasticity. Plasticity would thus be the mechanism by which each brain is unique, giving place for a materiality of singularity. They note, however, that the traits left by the lived experiences can be lasting or even permanent. Plasticity, according to the authors, is not synonymous with flexibility or permanent adaptability, it also constitutes a certain determinism that gives each individual a destiny that is his own.

The cognitive scientist Stanovich (2004) describe in the book Robot’s Rebellion: Finding Meaning in the Age of Darwin, that the evolutionary processes that gave birth to humans reached such a high degree of complexity, especially neural complexity, that two interrelated results occurred: first the brain was modeled in a highly elaborated anatomical differentiation, from a plurality of constituent elements that are not synchronized with each other, often leading to intracerebral conflicts, in which different parts present incompatible functions. Secondly, these intracerebral conflicts – understood as a materialized result of the sedimentation of several distinct periods of evolutionary history in the human central nervous system – make possible something unique to humans, which Stanovich calls “rebellion” against nature, insofar that it makes us humans prone to evolve against genetic and evolutionary determinism. According to Stanovich’s perspective, the evolution shaped human beings as vehicles capable of transmitting genetic material, with incredibly elaborate and flexible intelligence, also involving a sensitive and receptive plastic brain, so that evolutionary-genetic deterministic control, in our case, would have been relatively loosened to the point of producing creatures that escaped the control of genes in a completely unpredictable way. From the perspective of genes as “blind” replicators, the high complexity of the human body/brain system, is a kind of double-edged sword: while it allows replication strategies that are broader and more sophisticated than those of other living beings, the exceptional complexity of humans gives birth to biological processes that are disruptive to the natural dictates of evolution as they are transmitted by genes.

The neuroscientist Antonio Damasio (Damasio, 2010), also assumes a perspective quite similar to Stanovich’s, formulating that in man, the emergence of consciousness and the creation of culture are a radical novelty in the history of evolution, for these often offer imperfect or even “rebellious” responses, many of which go against the dictates of nature’s own laws: “If nature can be considered as indifferent, unpredictable, unpredictable, then human consciousness creates the possibility of question the ways of nature. The emergence of human consciousness is associated with evolutionary developments in the brain, behavior, and mind that ultimately lead to the creation of culture, a radical novelty in the movement of natural history. The emergence of neurons, with the emergence of the diversification of behavior and the paving of the path to the mind, is a momentary event in the great trajectory. But the appearance of the conscious brain, capable of self-reflection, is the next great moment. It is the opening of the way to a rebellious and imperfect response to the dictates of a careless nature” (Damasio, 2010, p.287).

As Adrian Johnston argues, the biological sciences need to be able to detach themselves from the idea of organicity (in the sense of a complete and harmonic whole) to conceive the brain in a way that is more compatible with the richness and complexity of human beings as speaking beings, as beings carrying within themselves something more than the organic. For him, to do justice to the rich and unpredictable kind of subject humans beings are, life sciences should complement the worldview of their spontaneous organicism with the notion that there is something more in the organic than the organic itself (Johnston, 2013). However, this non-organicity of the human brain should not be understood simply as equivalent to the inorganic. Johnston proposes that we call “anorganic” this non-organicity that is distinct from the inorganic. Unlike the inorganic, the term anorganic, as Johnston conceives it, designates the flaws in the organic structure and the dynamics engendered in and by the non-whole biological systems. For Johnston, scientific findings as those described above, show that the biomaterial substance of evolution seems to reflexively negate its own controls and causal influences, giving rise to beings whose complex plasticity escapes governance evolutionary-genetic nature.

## Conclusion

Since the 1990s psychoanalysis has been constantly criticized for having moved away from traditional methodologies of scientific investigation and, more specifically, from the emerging neuroscientific field. Lacanian psychoanalysis, which is very influential in the psychoanalytic field nowadays, tend to have a more critical position regarding the neurosciences, although some Lacanian psychoanalysts have done very interesting works in the interface between psychoanalysis and neuroscience.

One important conceptual underpinning for the rarity of debates with neuroscience in Lacanian psychoanalysis is the notion that psychoanalysis and sciences deal with different conceptions of the real. Lacan developed from Koyré the conception that the real with which science deal is a real entirely governed by the laws of physics and by the end of his teaching he states that the real for psychoanalysis is a real that conveys the absence of laws for sexuality - a ”real without law”.

We tried to show in this article that a new conception of the real is emerging from contemporary neurosciences, making possible new perspectives for the dialog between Lacanian psychoanalysis and neurosciences. A famous phrase from Ansermet and Magistretti state: “the individual can be considered biologically determined to be free, that is, to constitute an exception to the universal that carries him” (Ansermet and Magistretti, 2004, p.10). This only can happen because nature itself is fragile, vulnerable, subject to failures in its own materiality. This perspective challenges the most common intuitive view, in which nature is conceived as an omnipotent monolithic block composed of elements perfectly connected and controlled by deterministic laws. The more recent discoveries of life sciences, and neurosciences in particular, makes possible to conceive the materiality of nature as prone to contingency, error, and complexity, which is a vision of the real that is closer to the Lacanian idea that the real is without law, that nature and brain are less deterministic than science previously thought. This opens up an ontological and epistemological space for new debates between Lacanian psychoanalysis and neurosciences.

When the proposal for an approximation between psychoanalysis and neurosciences was initially proposed in the 1990s, some authors saw this approach as a project that aimed to adapt psychoanalysis to traditional methods of empirical investigation and sought to integrate psychoanalysis into science. I tend to agree with Bazan (2011) when she says that psychoanalysis owes to clinics its fundamentals and originality, and it is from this position that psychoanalysis can contribute to the neuroscientific debate about the fundamentals of consciousness and subjectivity, endowed with its different but nevertheless elaborated and systematized theory. As Bazan says this should also constitute a fundamental basis to be taken seriously. But answering to those who are skeptical about this approximation and who ask what would be the gain for psychoanalysis with this kind of approach, Bazan’s response seems perfect to me: “I do not know. There is no agenda for what there is to win, nor, for that matter, for what there is to lose. Rather, it is the game itself, the sole faithfulness to something that is happening, which should be decisive. Something is happening, which clearly and loudly summons psychoanalysis to respond; as said, how to respond, is not *a priori* clear, but a non-response would imply a certain loss.” (Bazan, 2011, p. 3).

## Author Contributions

The author has researched and wrote the article.

## Conflict of Interest Statement

The author declares that the research was conducted in the absence of any commercial or financial relationships that could be construed as a potential conflict of interest.
